# Joint Statement of the Korean Society for Preventive Medicine and the Korean Society of Epidemiology on the response to the COVID-19 outbreak

**DOI:** 10.4178/epih.e2020018

**Published:** 2020-04-29

**Let us focus our entire social capacity on preemptive checks and preparations to prevent community spread**

A coronavirus infection was identified as the cause of an outbreak of pneumonia originating in Wuhan, China. As of February 4, 2020, approximately a month after the epidemic was recognized, there have been 20,000 confirmed cases and 420 deaths. In Korea, as of February 4, 16 cases have confirmed, and hundreds are either in self-isolation or under active monitoring.

To date, the government has raised its infectious disease alert level to the second-highest level. South Korea has been doing well regarding quarantine, including the management of risk groups and contacts. However, as the prevalence of infections in China and other countries continues to increase, there is still a danger of community spread and the safety of our people is under serious threat. The current quarantine and symptom-based management system is limited and it seems that community spread is inevitable.

In this regard, based on the evidence on possibility of an incubation period (asymptomatic infection), we suggest the following recommendations as precautionary measures to minimize public health damage caused by the current influx of COVID-19 as well as to prevent community spread.

First, it is recommended to perform preemptive checks and prepare to prevent community spread considering the phase of infection and spread, and patterns of close and casual contact with domestically confirmed patients in China. This includes the recommendation for two weeks of self-quarantine and active cooperation in epidemiological investigations from anyone returning from countries at risk of coronavirus.

Second, in order to perform early detection and prevent the community spread, it is recommended that people, including foreigners who have visited China within the last two weeks or have been in contact with confirmed patients, who have been diagnosed with fever and respiratory symptoms, visit medical facilities available to perform testing and consult a local public health center or the Korea Centers for Disease Control and Prevention (KCDC). Furthermore, it is recommended that the intensive management of all domestic and foreign nationals entering from high-risk countries, including China, be taken into account in consideration of the spread and control of new coronavirus infections in China and neighboring areas.

Third, in case the spread of the COVID-19 to the local community becomes clear, it is recommended to establish close cooperative governance as soon as possible between the central government’s quarantine authority centered on the KCDC and the defense organizations of each local government (regional and basic municipalities). In addition, considering a situation where there is a lack of quarantine specialists, such as central epidemiologists, leading to the overburdening of the available specialists, it is recommended that specialists from the Infectious Disease Management Support Group (in case of uninstalled areas, the field epidemiology education colleges) be installed in each regional area, ensuring their public status and immediately putting them into epidemiological surveillance and quarantine services in each region.

Fourth, regarding the risk of spreading in the community and protecting the public’s right to health, it is recommended for people with respiratory symptoms to wear a mask, wash their hands with soap for more than 30 seconds, and cover their mouth with their sleeve when coughing. We strongly encourage cooperation with infection control measures, such as visitor restrictions, performed in medical institutions.

Fifth, in this public health crisis, the unified efforts and mutual trust of all sectors of society, including the general public, healthcare professionals, government authorities, and politicians, is critical to minimize and quickly overcome the potential human and social damage. Excessive fear not commensurate to the real danger leads to excessive contraction of daily activities, leading to unnecessary social costs. Thus, a mature response to this challenge is required. In addition, since both confirmed patients and self-quarantined individuals are victims and our neighbors, we ask for strong community solidarity that encourages and supports each other.

Finally, following the MERS outbreak in 2015, the public health crisis due to the outbreak of infectious diseases such as the new coronavirus infection can occur at any time. The importance of establishing an advanced defense infrastructure, such as a prepared system and the continuous training of professional personnel is emphasized. In addition to preventing the spread of COVID-19, the necessity to re-examine and supplement the preparedness and countermeasures system against foreign infectious diseases is urgent. The Korean Society of Preventive Medicine and Korean Society of Epidemiology will work with the government to respond to the COVID-19 outbreak, and prevent infectious diseases in the future by acting together with the people.

04 February 2020

The Korean Society for Preventive Medicine and the Korean Society of Epidemiology Emergency Committee on the Coronavirus

Disease 2019 (COVID-19) Outbreak

