# Supplementary material for: Joint Statement of the Korean Society for Preventive Medicine and the Korean Society of Epidemiology on the response to the COVID-19 outbreak
Source: Epidemiol Health. 2020 Apr 29;42:e2020018. doi: 10.4178/epih.e2020018 (PMC7285435; doi:10.4178/epih.e2020018)
Supplement: Supplementary file 1 [file epih-42-e2020018-suppl.pdf]

Volume: 42, Article ID: e2020018
https://doi.org/10.4178/epih.e2020018

신종코로나바이러스감염증 대응을 위한 대한예방의학회·한국역학회 공동 성명서

지역사회 유행 확산에 대비한 선제적 점검과 준비에 전 사회적 역량을 집중하자 !!

중국에서 시작한 신종코로나바이러스감염증은 유행이 인지된지 약 1 달만인 2020 년 2 월 4 일 현재 20,000 여명의 확진자와 420 여명의 사망자를 내면서 확산 중에 있다. 우리나라도 2 월 4 일 현재 16 명의 확진자에 수백명이 자가격리 또는 능동 관리상태로 놓여 있다.

현재까지 정부는 감염병 위기단계「경계」수준으로 상향하고 위험군 및 접촉자 관리를 포함한 방역 업무를 잘 해내고 있으나 국외에서의 유행이 장기화되면서 여전히 지역사회 전파의 위험은 남아있으며 우리 국민의 안전이 심각하게 위협을 받고 있다. 현재 검역과 증상에 기초한 관리방안 등은 제한적일 수 밖에 없고 지역사회유행을 피하기 어려울 것으로 보인다.

이에, 대한예방의학회·한국역학회 신종코로나바이러스 감염증 대책위원회에서는 잠복기(미증상 감염기) 전파 가능성의 근거가 제시된 것에 주목하며, 지금 진행 중인 신종코로나바이러스 유입에 의한 국민의 건강피해를 최소화 하고, 지역사회 전파 확산에 대한 사전 예방을 위하여 아래와 같은 권고안을 제안한다.

첫째, 현재 중국에서의 전파 확산 속도와 국내 확진 환자와의 밀접, 일상 접촉을 통한 전파양상 등을 고려해 지역사회 유행 확산에 대비한 선제적 점검과 준비를 본격화 할 것을 권고한다.

Volume: 42, Article ID: e2020018
https://doi.org/10.4178/epih.e2020018

여기에는 모든 위험지역에서 온 입국자들의 2 주간의 자발적인 자가격리 권고와 적극적인 역학조사 협조를 포함한다.

둘째, 신종코로나바이러스감염증 조기발견과 지역사회 확산방지를 위해, 최근 2 주 이내 중국을 방문했던 국민 및 외국인, 그리고 확진환자와 접촉했던 적이 있는 분 중 발열과 호흡기 증상이 있는 경우, 선별진료소가 있는 의료기관을 방문하거나, 관할 보건소, 질병관리본부 상담센터로 상담해줄 것을 권고한다. 나아가, 신종코로나바이러스감염증의 중국과 인근지역으로의 확산과 통제 상황을 감안해 중국을 포함해 고위험지역에서 입국하는 모든 내, 외국인에 대한 집중 관리를 적극 검토하기를 권고한다.

셋째, 신종코로나바이러스감염증의 지역사회 확산이 현실화할 경우를 대비해, 질병관리본부를 중심으로 한 중앙정부 방역당국과 각 지방정부(광역 및 기초지자체)의 방역조직간의 긴밀한 협력적 거버넌스를 조속히 구축할 것을 권고한다. 아울러, 중앙의 역학조사관 등 방역 전문인력이 부족하고 이들의 업무가 과중되고 있는 상황을 고려해, 각 광역지자체에 설치된 감염병관리지원단(미설치된 지역의 경우, 감염병관리 현장역학교육 담당 대학)의 전문인력을 민간역학조사관으로 공적 신분을 보장해, 각 지역의 역학조사와 방역업무에 즉각 투입할 것을 권고한다.

넷째, 지역사회에서의 전파 위험과 국민들의 건강권 보호를 위해 일상 생활에서 자주, 30 초 이상 비누로 손씻기와 기침할때 옷 소매로 가리기, 기침 등 호흡기 증상자는 반드시 마스크를 착용할 것을 권고하고, 의료기관에서 실시하고 있는 면회객 제한 등 감염관리 조치에도 적극 협조해 줄 것을 당부한다.

Volume: 42, Article ID: e2020018
https://doi.org/10.4178/epih.e2020018

다섯째, 아울러, 이와같은 공중보건위기상황에서 인명 피해와 사회적 피해를 최소화하고, 신속히 극복하기 위해서는 일반 국민, 보건의료전문가, 정부당국과 정치권 등 우리 사회 각 부문의 단합된 노력과 상호신뢰가 무엇보다 중요하다. 실재하는 위험을 넘어서 지나친 공포는 일상적 활동의 과도한 위축으로 이어지고, 이는 불필요한 사회적 비용을 초래하기에 이에 대한 성숙한 대응을 요구한다. 나아가, 기 확진자와 자가격리 중인 접촉자 등도 모두 피해자이자 우리의 이웃들이기에, 서로를 격려하고 지지해 주는 강한 공동체 연대의식을 발휘해 줄 것을 당부한다.

끝으로, 지난 2015 년 메르스에 이어 이번 신종코로나바이러스 감염증 같이 감염병의 집단 발병에 의한 공중보건위기 상황은 은 언제라도 닥칠 수 있다. 이에 대비해 준비된 시스템과 훈련된 전문 인력의 상시적 양성 등 선진화된 방역인프라 구축이 중요하다고 다시금 강조한다. 이번 신종코로나바이러스감염증의 확산 방지와 함께 해외 유입 감염병에 대한 대비 및 대응 시스템에 대한 전면 재점검과 보완이 시급히 필요하다. 대한예방의학회와 한국역학회는 이번 신종코로나바이러스감염증 유행 대응과 향후 감염병 예방에 정부와 함께 노력하고, 국민과 함께 실천하고자 한다.

2020 년 2 월 4 일

대한예방의학회· 한국역학회 신종코로나바이러스감염증 대책위원회
